# Probiotics and live biotherapeutic products aiming at cancer mitigation and patient recover

**DOI:** 10.3389/fgene.2022.921972

**Published:** 2022-08-09

**Authors:** Zelinda Schemczssen-Graeff, Marcos Pileggi

**Affiliations:** ^1^ Comparative Immunology Laboratory, Department of Microbiology, Parasitology and Pathology, Federal University of Paraná, Curitiba, Brazil; ^2^ Environmental Microbiology Laboratory, Structural and Molecular Biology and Genetics Department, Life Sciences and Health Institute, Ponta Grossa State University, Ponta Grossa, Brazil

**Keywords:** metabolomics, metagenomics, proteomics, epigenetics, chemical signaling, dysbiosis, gut microbiome, bacterial diversity

## Abstract

Molecular biology techniques allowed access to non-culturable microorganisms, while studies using analytical chemistry, as Liquid Chromatography and Tandem Mass Spectrometry, showed the existence of a complex communication system among bacteria, signaled by quorum sensing molecules. These approaches also allowed the understanding of dysbiosis, in which imbalances in the microbiome diversity, caused by antibiotics, environmental toxins and processed foods, lead to the constitution of different diseases, as cancer. Colorectal cancer, for example, can originate by a dysbiosis configuration, which leads to biofilm formation, production of toxic metabolites, DNA damage in intestinal epithelial cells through the secretion of genotoxins, and epigenetic regulation of oncogenes. However, probiotic strains can also act in epigenetic processes, and so be use for recovering important intestinal functions and controlling dysbiosis and cancer mitigation through the metabolism of drugs used in chemotherapy, controlling the proliferation of cancer cells, improving the immune response of the host, regulation of cell differentiation and apoptosis, among others. There are still gaps in studies on the effectiveness of the use of probiotics, therefore omics and analytical chemistry are important approaches to understand the role of bacterial communication, formation of biofilms, and the effects of probiotics and microbiome on chemotherapy. The use of probiotics, prebiotics, synbiotics, and metabiotics should be considered as a complement to other more invasive and hazard therapies, such chemotherapy, surgery, and radiotherapy. The study of potential bacteria for cancer treatment, as the next-generation probiotics and Live Biotherapeutic Products, can have a controlling action in epigenetic processes, enabling the use of these bacteria for the mitigation of specific diseases through changes in the regulation of genes of microbiome and host. Thus, it is possible that a path of medicine in the times to come will be more patient-specific treatments, depending on the environmental, genetic, epigenetic and microbiome characteristics of the host.

## Overview

The most obvious perception that people have about microorganisms since the 19th century is that they are mostly pathogens for humans. In more recent years, molecular biology techniques, mainly cloning and sequencing, brought to light the ideas of healthy microbiomes, while studies using analytical chemistry, as Liquid Chromatography and Tandem Mass Spectrometry, showed the existence of a complex communication system among bacteria, signaled by quorum sensing molecules. The ability to chemically communicate among host cells and their microorganisms was, then, the next scientific achievement, with the definition of the gut-brain axis microbiome. The development of metabolome and metagenomic, combined with traditional genetic and microbiological techniques, allowed the concept of dysbiosis to be developed, in which imbalances in the diversity of the human microbiome could lead to the constitution of different diseases, such as diabetes, allergies, autoimmune, autism, Alzheimer’s and cancer. Evolutionarily, microbiomes can be passed between generations, from mothers to children, considered as epigenetic inheritances since they can regulate the expression of genes in the hosts. The earlier and more effective this transference from mothers to children is, the more effective the epigenetic imprint can be. Advent such as the use of antibiotics, environmental toxins, processed foods, lead to the loss of diversity in the gut microbiome, causing dysbiosis. In this context, the use of probiotic bacterial strains, such as those of the *Lactobacillus* and *Bifidobacterium* genera, has the objective of recovering important intestinal functions and controlling dysbiosis. The understanding that the metabolites of these strains, and others not yet considered as probiotics, the next-generation probiotics, can have a controlling action in epigenetic processes, has enabled the use of these bacteria not only as food additives capable of improving general human health, but as Live Biotherapeutic Products, with the objective of mitigating specific diseases through changes in the regulation of microbiome and host genes. Colorectal is one of the most well-studied types of cancer in terms of its relationship microorganisms. The gut microbiome, in dysbiosis configuration, is involved with tumor by the biofilm formation, the production of toxic metabolites or inducing DNA damage in intestinal epithelial cells through the secretion of genotoxins. However, healthy gut microbiota has been used in therapeutic drugs metabolism for chemotherapy, radiotherapy response modulation, and targeted immunotherapy. There are distinct levels of epigenetic control by probiotic strains or Live Biotherapeutic Products possible to be envisioned in controlling the proliferation of cancer cells, as the inactivation ratios of cancerogenic compounds; improving the immune response of the host; antiproliferative effects*via*regulation of cell differentiation and apoptosis; inhibition of tyrosine kinase; and inflammatory cell infiltration among malignant and stromal cells. As an example, gut bacteria can metabolize fiber into butyrate, a short-chain fatty acid and a histone deacetylase inhibitor, that upregulates tumor-suppressor genes epigenetically in cancer cells and anti-inflammatory genes in immune cells. Not just in cancer, probiotic bacteria are also associated with DNA methylation and the induction of regulatory T-cells, that normally suppress inflammatory, opening the possibility of immunologic diseases treatment, as allergic and autoimmune disorders. A relationship between dysbiosis and microbiome can also be found for breast cancer. Both the gut microenvironment and breast tissue participate in this system. Epigenetics is associated with cancer development in postmenopausal women, through the regulation of steroid-hormone metabolism, mainly estrogens. In contrast, the gut resident microbiome can modulate mucosal and systemic immune responses. There are still gaps in studies on the effectiveness of the use of probiotics strains and Live Biotherapeutic Products in cancer treatments because the metabolic interrelationships among resident microbiome, environmental factors and genetic/epigenetic determinants of the vulnerable host are complex. Metabolome, metagenomics, transcriptome, and analytical chemistry are important approaches to understand the role of bacterial communication*viavia*quorum sensing, formation of biofilms, and the interference of microbiome and probiotics on chemotherapy. The metabolic and epigenetic interactions between colorectal cancer and resident microbiome are robust experimental model for studies in diverse types of cancer. The use of probiotics, prebiotics, synbiotics, and metabiotics should be considered as a complement to other more invasive and hazard therapies, such chemotherapy, surgery, and radiotherapy. Thus, it is possible that a path of medicine in the coming times will be more specific treatment for the patient, depending on the environmental, genetic, epigenetic and microbiome characteristics of the host. For this, it is necessary to have a better detail of the regulation of genes associated with specific tumors, of metabolites associated with down and up regulation of these genes, and, finally, which bacterial strains are candidates to produce these substances efficiently within the intestinal system.

## Literature review

### Microbiomes and host genes in chemical communication

For most people microorganisms are primarily pathogens for humans. This is a concept supported since the 19th century with the germ theory of disease and Koch’s postulates, therefore the view of the nascent discipline of microbiology focused on the pathogenic potential of microorganisms. A classic example of this postulate is the association of *Helicobacter pylori* with peptic ulcers recurrence and gastric cancer (Chen et al., 2019).

The concept of healthy microbiomes playing an important role in human physiology has been built more recently in the history of Science, thanks to molecular biology techniques, mainly cloning and sequencing. Strain-level differences in microbiomes has allowed a better understanding of disease associations, not only with cancer, but with a host of diseases, and this have been achieved with a more detailed study in metagenomic sequencing and, therefore, access to complete genomes, as was the case with *Propionibacterium acnes,* being able to compare healthy skin microbiomes and those linked to acne, at strain level (Bangayan et al., 2020). Studies using analytical chemistry showed the existence of a complex communication system among bacteria, signaled by quorum sensing molecules, a population density-dependent characteristic, and that can allow adaptations to environmental conditions, such those involving bioluminescence, antibiotic biosynthesis, plasmid conjugation and virulence (Kiymaci et al., 2018). *Pseudomonas aeruginosa* is an example of an opportunistic human bacterium that causes devastating infections in patients with compromised immune systems, and its ability to form antibiotic-resistant biofilms it is probably the reason for the persistence in clinical settings (Thi et al., 2020). Novel approaches have shown the interference of quorum sensing molecules in other functions, such as coordinate response systems, as antioxidative enzymes production or biofilm formation to tolerate herbicides (Freitas et al., 2021). There is great diversity in communication networks mediated by quorum sensing signals, including in virulence modulation, therefore have become a promising target for mitigating pathogens (Contreras-Ramos and Mansell, 2021).

The ability to chemically communicate among host cells and their microorganisms was, then, the next scientific achievement, with the definition of the gut-brain axis microbiome, which can be defined as the bidirectional chemical communication among the gut, its microbiome, and the nervous system (Bengesser et al., 2019; Osadchiy et al., 2019; Doenyas, 2021). The understanding of communication exclusively between bacteria through quorum sensing signaling molecules possibly inspire studies of communication between bacteria and their eukaryotic hosts, which be conducted through other signaling molecules such as butyrate, propionate, and acetate, related to epigenetic and cancer. These relationships will be exemplified throughout this review. Intestinal neurons can sense bacteria independently of the host immune system, as the mediators with neuromodulatory properties produced by *Staphylococcus aureus*, which increase the membrane permeability in cultured sensory neurons, and change intestinal motility and secretion through the induction of biphasic response in extrinsic sensory afferent nerves (Uhlig et al., 2020). The dysfunction of the brain-gut-microbiome axis is the most important etiological factor for the irritable bowel syndrome, with the neurotransmitter serotonin taking a particularly significant role in the pathology. Thereby, susceptible genes for this disease are related to serotonergic signaling pathways (Mishima and Ishihara, 2021). Long-term treatment with multispecies probiotics attenuated the memory dysfunction through the decreased of trimethylation of histone H3 Lys 27 in lead-exposed rats (Xiao et al., 2020).

The development of metabolome and metagenomic, combined with traditional genetic and microbiological techniques, allowed the concept of dysbiosis to be developed, in which imbalances in the human microbiome diversity could lead to the constitution of different diseases. The identification of obesity-associated gut microbial species, as the glutamate-fermenting commensal *Bacteroides thetaiotaomicron*, was achieved by metagenome sequencing and serum metabolomics profiling in a cohort of lean and obese (Liu et al., 2017). Primary bile acids, through bacterial metabolism, produces secondary bile acids, as in *Ruminococcaceae* and is associated with ulcerative colitis in colectomy-treated patients (Sinha et al., 2020). Gut microbiota dysbiosis can be associated with chronic heart failure. Particularly, *Faecalibacterium prausnitzii* was found at lower population levels and *Ruminococcus gnavus* was found higher in affected patients’ gut microbiota than in controls (Cui et al., 2018). Parkinson’s disease can be characterized by the accumulation of intracellular aggregates of misfolded *a*-synuclein along the cerebral axis, and this was associated with a reduction of bacteria *Butyrivibrio*, *Pseudobutyrivibrio*, *Coprococcus*, and *Blautia*, belonging to the Lachnospiraceae family, linked to anti-inflammatory/neuroprotective effects (Vascellari et al., 2020). The increased risk of colorectal cancer is associated with dietary fat intake, with the increase of specific strains of bacteria *Alistipes* sp. And gut metabolite alteration, including elevated lysophosphatidic acid, which promotes cell proliferation and impair cell junction (Yang et al., 2022).

**FIGURE 1:**
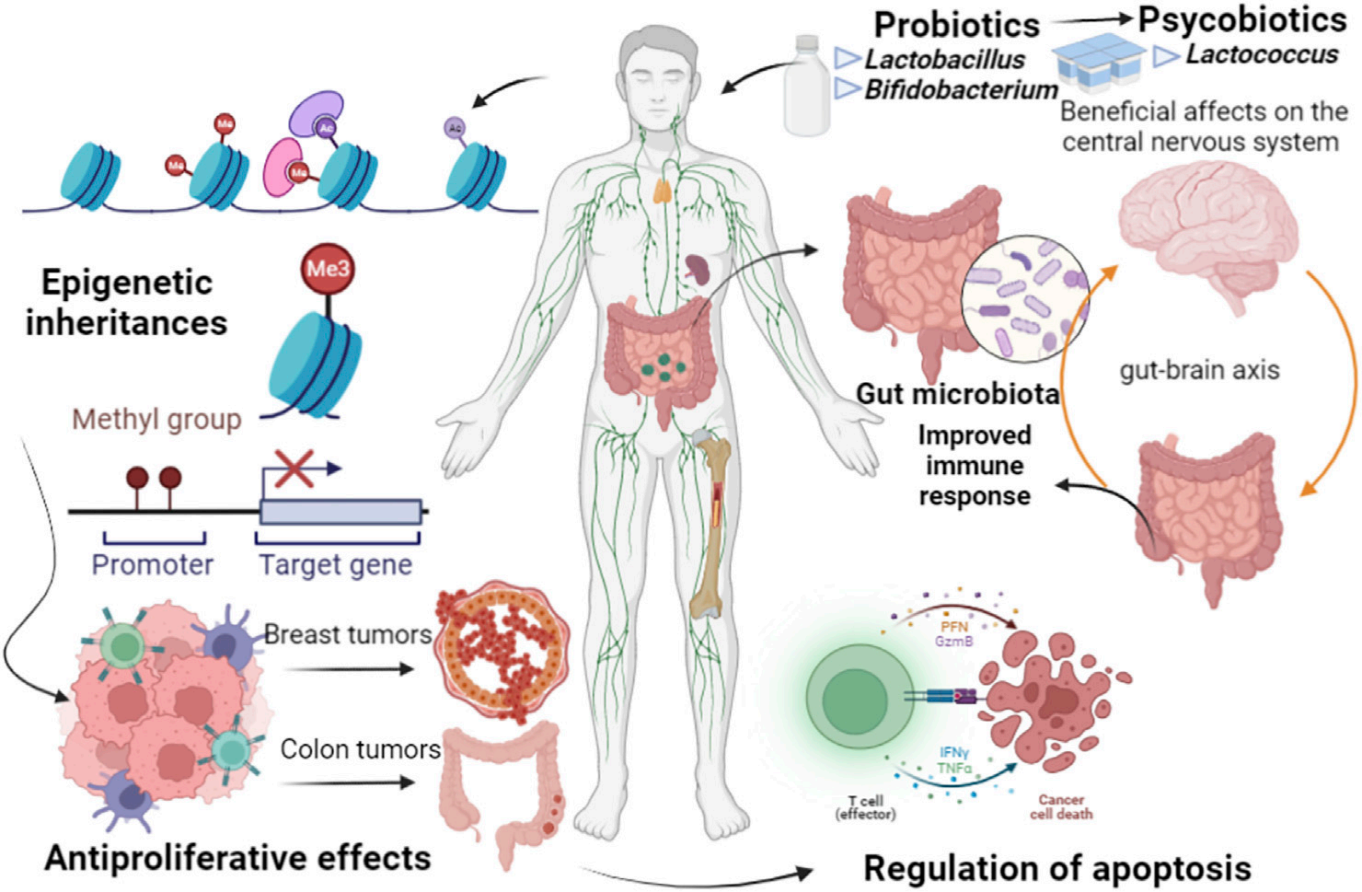
Epigenetic inheritances can be passed among host generations, regulated by gut microbiome. Several factors can cause dysbiosis, characterized by changes in microbiome diversity, related to initiation and promotion of chronic inflammatory pathways, promotion of genetic and epigenetic alterations, leading to tumor origin and development, as in breast and colorectal cancer. Nevertheless, probiotic bacteria, as *Lactobacillus* and *Bifidobacterium,* can induce specific mechanisms against various infections, including diverse types of cancer, through apoptosis, antioxidant activity, immune response induction, and epigenetics regulation.

Evolutionarily, microbiomes can be passed among generations, from mothers to children, considered as epigenetic inheritances since they can regulate the expression of genes in the hosts (Figure 1). The earlier and more effective this transference from mothers to children is, the more effective the epigenetic imprint can be. Neonatal supplementation in adult mice with p40, a probiotic functional factor, protect the gut against inflammation, and protection against colitis, through an epigenetic imprint on anti-transforming growth factor *ß* TGFβ, leading to long-lasting production by intestinal epithelial cells to expand Tregs (Deng et al., 2021).

### Dysbiosis, probiotics, epigenetics in colorectal cancer

The metabolic and epigenetic interactions between colorectal cancer and resident microbiome are well-studied, even because gut microbiomes are most studied so far, able to serve as an experimental model for diverse types of cancer and other diseases. Advent such as the use of antibiotics, environmental toxins, processed foods, lead to the loss of diversity in the gut microbiome, causing dysbiosis, which can be related to several diseases, from obesity to colorectal cancer. Gut-derived lipopolysaccharide, gut microbiota-associated bile acids, tryptophan, and short-chain fatty acids are dynamically involved in liver regeneration after partial hepatectomy. However, these processes depend on the composition of gut microbiota, which was molded by antibiotics or probiotics (Zheng and Wang, 2021). The interaction between microbiota and the genetic processes that cause the tumors can be exemplified by serrated adenocarcinoma and sporadic colorectal carcinoma, which have histological and molecular characteristics of microsatellite instability and are associated with changes in methylation patterns, whose specificity in epigenetic regulation may help define the key molecules responsible for the weak immune response in these tumors, and identify potential targets for treatment. The work by García-Solano et al., 2018 validated HLA-DOA and CD14 in DNA, mRNA and protein level, as CD14 is related to the mediation of the innate immune response induced by bacterial lipopolysaccharide, while HLA-DOA is found in lysosomes in B cells and peptide regulation mediated by HLA-DM loading on MHC class II molecules. In this context, the use of probiotic bacterial strains, such as those of the *Lactobacillus* and *Bifidobacterium* genera, has the objective of recovering important intestinal functions and controlling dysbiosis (Figure 1). High-fat/carbohydrate diet in obesity cases is characterized by a gut microbiota with a predominance of Firmicutes (*Clostridium*), *Prevotella* and *Methanobrevibacter* but deficient in beneficial bacteria such as *Bacteroides*, *Bifidobacterium*, *Lactobacillus* and *Akkermansia* (Amabebe et al., 2020). Treatment with B6 vitamin and probiotic strains, as *B. longum* and *L. rhamnosus*, may alleviate symptoms in lactose intolerant patients through the increase in acetic acid, 2-methyl-propanoic acid, nonenal, and indolizine 3-methyl, and decrease in phenol (Vitellio et al., 2019). *Lactobacillus*, *Lactococcus* and *Bifidobacterium* can improve the host’s immune system through the regulation of brain pathways and serotonin production (Magalhães-Guedes, 2022). Infections caused by *C. butyricum*, *C. difficile*, and *C. perfringens* may originate in conditions of host dysbiosis. *L. plantarum* can be used to restore microbiota after antibiotic treatments to eliminate those opportunist bacteria (Monteiro et al., 2019).

The symbiotic relationship between eukaryotes and microbiomes has been described and might explain some transgenerational inheritance. The epigenetic modulation occurs from the first years of life, occurring even in adulthood, and can be altered with changes in eating habits and the use of antibiotics, which can lead to dysbiosis (Flandroy et al., 2018). The understanding that the metabolites of microbiomes can repair these conditions, the use of strains such as probiotics and others not yet considered as probiotics, the next-generation probiotics, can have a controlling action in epigenetic processes, has enabled the use of these bacteria not only as food additives capable of improving general human health, but as Live Biotherapeutic Products, with the objective of mitigating specific diseases through changes in the regulation of Microbiome and host genes. The Food and Drug Administration of US Government has defined the category of “live biotherapeutic products” and constituting objectives and regulations for pharmaceutical uses and quality requirements, so these products could reach the market and registered as medicinal products (Cordaillat-Simmons et al., 2020).

Epigenetic mechanisms driven by gut microbiome are more relevant in early childhood and related to the type of delivery, breastfeeding, introduction of solid food, infections, and antibiotic treatments. Short-chain fatty acids, produced by fermentative metabolism of gut microbiome can inhibit histone deacetylase activity and modulate host gene expression involved in cellular lipid metabolism and satiety, may lead to obesity. These are characteristic situations of reduced microbial diversity. The presence of abundant Firmicutes in pregnant women produce a pattern of differentially methylated promoters, which is associated to lipid metabolism, inflammatory response, and risk of obesity (Cuevas-Sierra et al., 2019).

**FIGURE 2:**
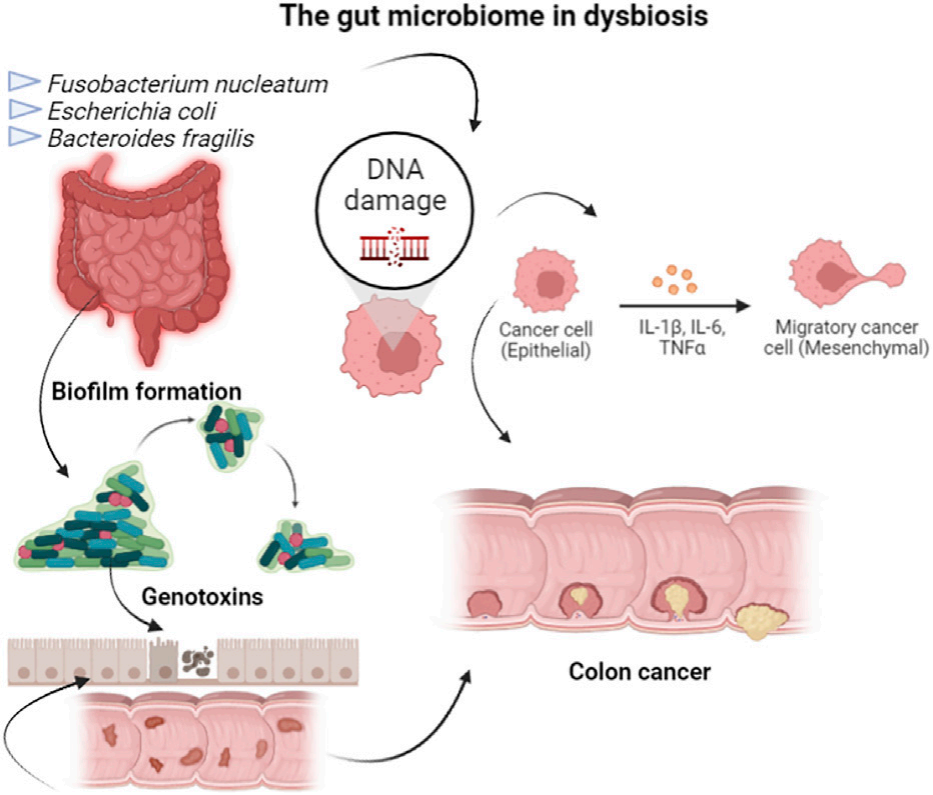
Bacteria, as *F. nucleatum* and specific strains of *E. coli* and *B. fragilis*, in dysbiosis configuration of the gut microbiome, is involved with colorectal cancer by the host epigenetic regulation, biofilm formation, toxic metabolites production, DNA damage induction in intestinal epithelial cells through the secretion of genotoxins, or induction of T-cell-mediated immune responses against colorectal tumors.

Colorectal is one of the most well-studied types of cancer, being that surgery, radiotherapy, and chemotherapy are the normally used treatments, but presenting side effects as systemic toxicity, resistance, and recurrence (Sharma and Shukla, 2016). Colorectal cancer is also well-studied in terms of its relationship with microorganisms. *Fusobacterium nucleatum* and specific strains of *Escherichia coli* and *Bacteroides fragilis* were related to colorectal carcinogenesis, using DNA sequencing and functional studies in animal models (Wong and Yu, 2019). The gut microbiome, in dysbiosis configuration, is involved with tumor by the biofilm formation, the production of toxic metabolites or inducing DNA damage in intestinal epithelial cells through the secretion of genotoxins. However, healthy gut microbiota, or the use of probiotics strains, has been related to drugs metabolism used in chemotherapy, in modulation of radiotherapy responses, and targeted immunotherapy. For example, dysbiosis can enhance the amount of *F. nucleatum*, which was associated with high microsatellite instability and methylation status of proto-oncogen BRAF, consequently promoting colorectal tumor growth through the inhibition of T-cell-mediated immune responses. This situation results in shorter survival of cancer patients (Figure 2). In contrast, mice submitted to therapy based on anti-programmed cell death 1 and then treated with probiotics showed a lower frequency of interferon-γ-positive cytotoxic T cells in the tumor microenvironment (Spencer et al., 2021). Cancer prevention and treatment strategies can be achieved through diet, probiotics, and antibiotics (Mima et al., 2016). A study conducted in colorectal cancer patients with colon resection showed that *Saccharomyces boulardii* deregulated pro-inflammatory cytokines (Consoli et al., 2016).

Loss of beneficial microorganisms is a crucial element for the origin of dysbiosis, in which pathogens and pathobionts have harmful configurations for hosts in different constituents of microbiome diversity (Silva et al., 2021). Therefore, dysbiosis may be related to initiation and promotion of chronic inflammatory pathways and promotes the colorectal cancer through genetic and epigenetic changes, which lead to dysplasia, clonal expansion, and cell malignant transformation (Figure 1).

Probiotic bacteria can mitigate the initiation of the carcinogenic process and the effects of the already established disease, through its metabolism and chemical communication, with systemic effects on the host, as cancerogenic compounds inactivation; competition with putrefactive and pathogenic microbiome; improvement of the immune response of the host; antiproliferative effects *via* regulation of cell differentiation and apoptosis; undigested food fermentation; tyrosine kinase inhibition; reduction of enteropathogenic complications related to colon cancer surgery. Probiotics strains can also improve diarrhea but affecting peristalsis and, therefore, the efficient functioning of the intestine and promoting the integrity of gut mucosal. This chain of effects culminates with the stimulation on the immune system and prevention of the metastasis in colorectal cancer (Eslami et al., 2019). Microbiome, probiotic strains, and colorectal cancer can be linked through quorum sensing, biofilm formation, sidedness, and effects/counter effects on chemotherapy. Studies on genomics and metabolomics targeting the gut microbiome will uncover important linkages between microbiome and intestinal health (Raskov et al., 2017). Gut microbiome, depending on the bacterial biodiversity, can secrete genotoxins that lead to DNA damage in intestinal epithelial cells and initiate a tumor. The efficacy of chemotherapy, radiotherapy and immunotherapy also will depend on microbiome diversity, and this may vary from patient to patient (Silva et al., 2021). This specific host-microbiome relationship creates an opportunity to control the health of an individual by manipulating the composition of the gut microbiota, which can be achieved through the administration of probiotics, prebiotics, symbiotics, fecal microbiota transplantation, but with generic effects. Thus, new commensal strains, or the next-generation probiotics, have been sought as promising prophylactic and therapeutic agents (Singh and Natraj, 2021). In my opinion, this is the foundation of a new philosophy of patient-specific precision medicine. In addition to these approaches, metabiotics, or probiotic derived factors, which have epigenetic, antimutagenic, immunomodulatory, apoptotic, and antimetastatic effects, can optimize host physiological functions and be used in immunosuppressed individuals (Sharma and Shukla, 2016). Gut bacteria can be exploited in other therapies, such as immune cell boost, and oncolytic bacteria (Silva et al., 2021). One of these approaches is possible with the bacteria *B. animalis*, whose growth is stimulated by the dietary herbal medicine *Gynostemma pentaphyllum*, which has a prebiotic action in this case. This bacterium has enhanced expressions of genes encoding for biogenesis and metabolic pathways of short-chain fatty acid and medium-chain fatty acids, and these molecules are related to host responses in different cellular processes, as RNA processing, *a*-amino acid biosynthesis and metabolism, anion transmembrane activity, and transferase activity. These connected actions reduce polyps in laboratorial mice affected by colorectal cancer (Liao et al., 2021). Another possibility is engineered bacterial immunotherapy, using natural or engineered bacterial strains to deliver antitumor products or drugs, enhance both adaptive and innate immunity. For example, bacterial outer membrane vesicles can generate anticancer cytokines with few side effects (Zhou et al., 2021). In any case, there is still much to be studied in this area. For example, although the modulation of the immune system, targeting neoplasms, by the gut microbiome has improved the survivability of many individuals, predicting post-therapy outcomes is still difficult due to the insufficiency of predictive biomarkers. Thus, the study of the structure of bacterial species associated with tumors and their recovery, and inflammatory indicators,*viavia*metagenomics, is fundamental in these predictive studies. *Bifidobacterium* spp. for example, has been used efficiently for treating susceptibility to colitis after treatment with immune checkpoint inhibitors (Schwartz et al., 2019). In addition to *Bifidobacterium* spp. such as *B. pseudolongum*, *L. johnsonii* and *Olsenella* spp. they are related to the significantly increased efficacy of immune checkpoint inhibitors in colorectal cancer mouse models (Wu et al., 2021).

The use of probiotics, prebiotics, synbiotics, and metabiotics should be considered as a complement to other more invasive and hazard therapies, such chemotherapy, surgery, and radiotherapy. 5-fluorouracil is an important drug used in systemic chemotherapy treatment for colorectal cancer, but this therapy has been compromised by the development of chemoresistance, probably due to genetic and epigenetic factors of the patients. *L. plantarum* produces gamma-aminobutyric acid and GABAB receptor-dependent signaling pathway, which can be used as a treatment option for 5-fluorouracil-resistant cells because gamma-aminobutyric acid activates antiproliferative, anti-migration, and anti-invasion effects on the resistant cells. Activated GABAB receptor induces the inhibition of cAMP-dependent signaling pathways and cellular inhibitor of apoptosis protein 2 expression. This system has a predictive biomarker, adjuvant treatment for chemotherapy-resistant cancer cells, chemoprevention, and colon cancer-related diseases treatments potential (An et al., 2021).

Western diets influence colorectal cancer through the modulation of the composition and function of gut microbiome, which can produce oncometabolites or tumor-suppressive metabolites depending on the characteristic of gastrointestinal tract. Energy metabolites for the gut microbiome are essential cofactors for epigenetic enzymes, and they come from the available food to the hosts. Transcriptome profiles can show aberrant epigenetic marks that accumulate during colorectal cancer, indicating the epimutations that drive tumorigenesis. Nevertheless, healthy eating habits, as through dietary fiber, allow them to be metabolized by colonic bacteria into butyrate, short-chain fatty acid and histone deacetylase inhibitor. Butyrate epigenetically upregulates tumor-suppressor genes in colorectal cancer cells and anti-inflammatory genes in immune cells (Bultman, 2017). Anti-cancer drugs, based on histone deacetylase inhibitors, could inhibit tumor cell proliferation or apoptosis. Acetylation marks could be eliminated by the influence of gut microorganisms, featuring an essential epigenetic change in cancer cells (Salek Farrokhi et al., 2020). Patients with active Celiac Disease, carriers of the HLA DR3/DQ2 or HLA DR4/DQ8 haplotypes, show significant increases in the gene expression of several members of the NOD-like receptor family in a gluten-free diet. The regulator of these receptors, NLRX1, was exclusively down-regulated during active disease, allowing for inflammation-induced dysbiosis. These changes were accompanied by changes in the production of short- and medium-chain fatty acids (Morrison et al., 2022). Food and plant extracts that were fermented by gut microbiome, producing short-chain fatty acids, which act in epigenetics, immunological and molecular signaling pathways, thus playing protective role in colorectal cancer (Shuwen et al., 2019). Colorectal cancer has a genesis related to genetic predisposition and epigenetic events, under heavy influence of gut microbiome. Thus, probiotics, prebiotics and symbiotics can have a potentially positive effect on modulate the host inflammatory response and prevention and treatment of tumor proliferation, metastasis, and cancer inhibition. Galdeano and Perdigon, 2006 highlighted the importance of dosing the use of probiotics, about 10^8^ colony forming unit-CFU/day, and the time of intestinal permanence, ranging between 48 and 72 h, characteristics that optimally induce immunostimulation in the host. Metabolites produced in cancer cells environment can induce a chronic inflammatory response by the inflammatory cells and then the predisposing condition for cancer retention. The chronic inflammatory condition is strongly modulated by diet and gut microbiome (Almeida et al., 2019). The next-generation whole-genome sequencing, transcriptome sequencing, and big-data mining pharmacogenomics approaches can make it prospect for new experimental trials specifically for each patient, considering the clinical and pathological history. Therefore, only positive results could be obtained from the administration of probiotics individually for patients, based on their genetic structure, lifestyle, and environmental particularities and seeking to recover the host microbial homeostasis (Vivarelli et al., 2019; Sehrawat et al., 2021).

### Dysbiosis, probiotics, epigenetics in diverse types of cancer

There are various levels of epigenetic control by probiotic strains or Live Biotherapeutic Products possible to be envisioned in controlling the proliferation of cancer cells. Gut bacteria can metabolize fiber into butyrate, a short-chain fatty acid and a histone deacetylase inhibitor, that epigenetically upregulates tumor-suppressor genes in cancer cells and anti-inflammatory genes in immune cells.

Supplementation with bacterial mixture composed by *B. longum*, *B. breve*, *B. infantis*, *L. acidophilus*, *L. plantarum*, *L. casei*, *L. bulgaricus*, and *Streptococcus thermophilus*, changed the gut bacterial composition, the abundance of *Lachnospiraceae*, *Streptococcus*, and *Lachnoclostridium*, and could attenuate lung metastasis of melanoma in mice. These effects were achieved through the production of short-chain fatty acids in the gut, as propionate and butyrate, which promote the expression of chemokine ligand 20 in lung endothelial cells and the recruitment of T helper 17, decreasing the number of tumor foci in lungs (Chen et al., 2021).

Not just in cancer, probiotic bacteria are also associated with DNA methylation and the induction of regulatory T-cells, that normally suppress inflammatory, opening the possibility of treatment of allergic and autoimmune disorders, for example. *B. longum* subsp. *Infantis* and *L. rhamnosus* with DNA methylation properties, for example, have a regulatory T cells-inducing capacity, that normally suppress inflammatory events. The methylated CpG oligodeoxynucleotide from *B. longum* could be used as therapeutic vaccine for treating of immunologic diseases, such as the allergic and autoimmune disorders, in which Treg populations are diminished (Li et al., 2020).

A relationship between dysbiosis, microbiome and epigenetic reprogramming can also be found for breast cancer. Both the gut microenvironment and breast tissue participate in this system. In postmenopausal women, an important risk factor for the breast cancer development is the regulation of steroid-hormone metabolism, as estrogens, by the gut microbiome. Thus, diet, probiotics and prebiotics could affect the metabolism of drugs used in immunogenic chemotherapy, which may have an anticarcinogenic action. The gut microbiome produces low molecular weight bioactive substances such as folates, short-chain fatty acids, as butyrate and acetate, and biotin, and contributes to absorption and excretion of zinc, iodine, selenium, cobalt, and others, which are cofactors of enzymes participating in epigenetic processes (Figure 1). For example, butyrate to activate epigenetically silenced genes in cancer cells such as p21 and BAK. In view of these epigenetic processes, probiotic strains can be used in the mitigation of breast cancer, promoting effects in the immune response, leading also to breast tumor cell inhibition (Laborda-Illanes et al., 2020). Epigenetics is possibly associated with the regulation of steroid-hormone metabolism in postmenopausal women, as estrogens, for example, and then be involved in cancer development. In contrast, the gut resident microbiome can modulate mucosal and systemic immune responses.

There are still several gaps in studies on the effectiveness of the use of probiotics strains and Live Biotherapeutic Products in cancer treatments because the metabolic interrelationships among resident microbiome, environmental factors and genetic/epigenetic determinants of the vulnerable host are complex. *Lactobacillus* and other probiotic bacteria can induce specific mechanisms against various infections including cancers through apoptosis, antioxidant activity, immune response, and epigenetics regulation (Figure 1). *L. acidophilus* and *B. longum*, for example, are capable to reduce diarrhea after radiation treatment in cancer patients (Demers et al., 2014). However, further investigations must analyze more data to show the efficiency and type of contribution in mitigating diverse types of cancer using probiotics in clinical practice (Dasari et al., 2017; Drago, 2019). There is no consensus among researchers about the human commensal microbiome is a key determinant in the etiopathogenesis of cancer, but large longitudinal, cohort studies should be a future research priority. However, the microbiome, the environmental factors, and an epigenetically/genetically vulnerable host, indicates that multidirectional interactome drives carcinogenesis (Scott et al., 2019). A reliable source of information should be obtained through the metabolome, metagenomics, transcriptome, and analytical chemistry, which are important approaches to understand the role of bacterial communication and the effects of microbiome and probiotics on cancer chemotherapy. For example, the role of the gut microbiome in epithelial tumors, including non-small cell lung, kidney cancer, and melanoma, is done by blocking anti-programmed cell death. Protein 1 or the ligand of these immunotherapy drugs since the use of broad-spectrum antibiotics was associated with the immunotherapy failure. Another example is the fecal microbiota transplantation in mice, in which the level of antitumor CD8^+^ T cells was increased in immunotherapy responders, while the level of immunosuppressive CD4^+^ T cells was lower in non-responder’s mice (Wan et al., 2021).

### Epigenetics mechanistic approaches of probiotics for colorectal cancer mitigation

The effects of using probiotic strains and gut microbiomes in preventing human health are well known and several articles have been published on this subject. These are effects on the immune system, host metabolism, nutrient absorption and vitamin synthesis, increased resistance to opportunistic strains and production of short-chain fatty acids, which are crucial in epigenetic systems, the subject of this review. It is necessary to make it clear that the antitumor mechanisms presented by probiotics are not fully understood.

Different species of *Lactobacillus* are related to upregulate the B-cell lymphoma 2-associated X/B-cell lymphoma 2 ratio, increasing apoptosis in colon cancer cell lines. *L. casei* is also involved with an apoptosis through the upregulated expression of the tumor necrosis factor -related apoptosis-inducing ligand, which was induced by tumor necrosis factor *a*-mediated apoptosis. *L. paracasei* subsp. *Paracasei* and *Bacillus polyfermenticus* can reduce the expression of genes from the cyclin group of cell cycle regulators, associated with tumor development. The use of the probiotic *L. plantarum* inhibits the development of gastric cancer cell lines through the downregulation of the Murine Thymoma Viral Oncogene and upregulation of the phosphatase and tensin homolog, B-cell lymphoma 2-associated X, and toll-like receptor 4. Different species of *Lactobacillus* inhibit the production of interleukin-8 and interferon gamma, attenuating inflammation in gastric epithelial cells and inhibit the adhesion of the bacterium *H. pylori*, linked to the initiation of gastric and colorectal cancer. (Davoodvandi et al., 2021).

Probiotics may play an important role in the prognosis of tumors, based on the concept that the intestinal microbiota can regulate the immune balance and the “tumor of an organic environment” (TOE), which is related to the tumor microenvironment, and which involves tumor cells, fibroblasts, intratumoral microorganisms and metabolites in the local lesion. TOE also involves the immunity, circulation, metabolism, and gut microbiota closely related to tumor development (Wu et al., 2021). In this context, not so much probiotic strains, but next-generation probiotics and live biotherapeutic products may play an important role in tumor prognosis since they have a more detailed genetic and metabolic relationship with their hosts.

### Epigenetics mechanistic approaches of probiotics for mitigation of diverse types of cancer

*L. plantarum* reduced the expression of mitogen-activated protein kinase in oral cancer cell lines, reducing homeostatic and pathologic sequelae caused by intracellular responses under the control of this enzyme. Still in oral cancer, *L. salivarius* can decrease the expression level of cyclooxygenase-2 and proliferating cell nuclear antigen, decreasing the effects of the disease. Another gene whose inhibition is important for decreasing the vascularization of tumor cells is ornithine decarboxylase, induced by *L. rhamnosus*. The probiotic strain *L. reuteri* can downregulate the expression level of the urokinase plasminogen activator/urokinase plasminogen activator receptor gene, which is related to the degradation of extracellular matrix components and to cancer metastasis and invasion. *B. longum* and *L. acidophilus* treatment of Barrett’s esophagus cells downregulated the expression of caudal type homeobox 1, cyclooxygenase-2, tumor necrosis factor α, and phosphoprotein. 53, while the expression level of Interleukin-18 was enhanced, inhibiting the proliferation of cancer cells (Davoodvandi et al., 2021).

**FIGURE 3:**
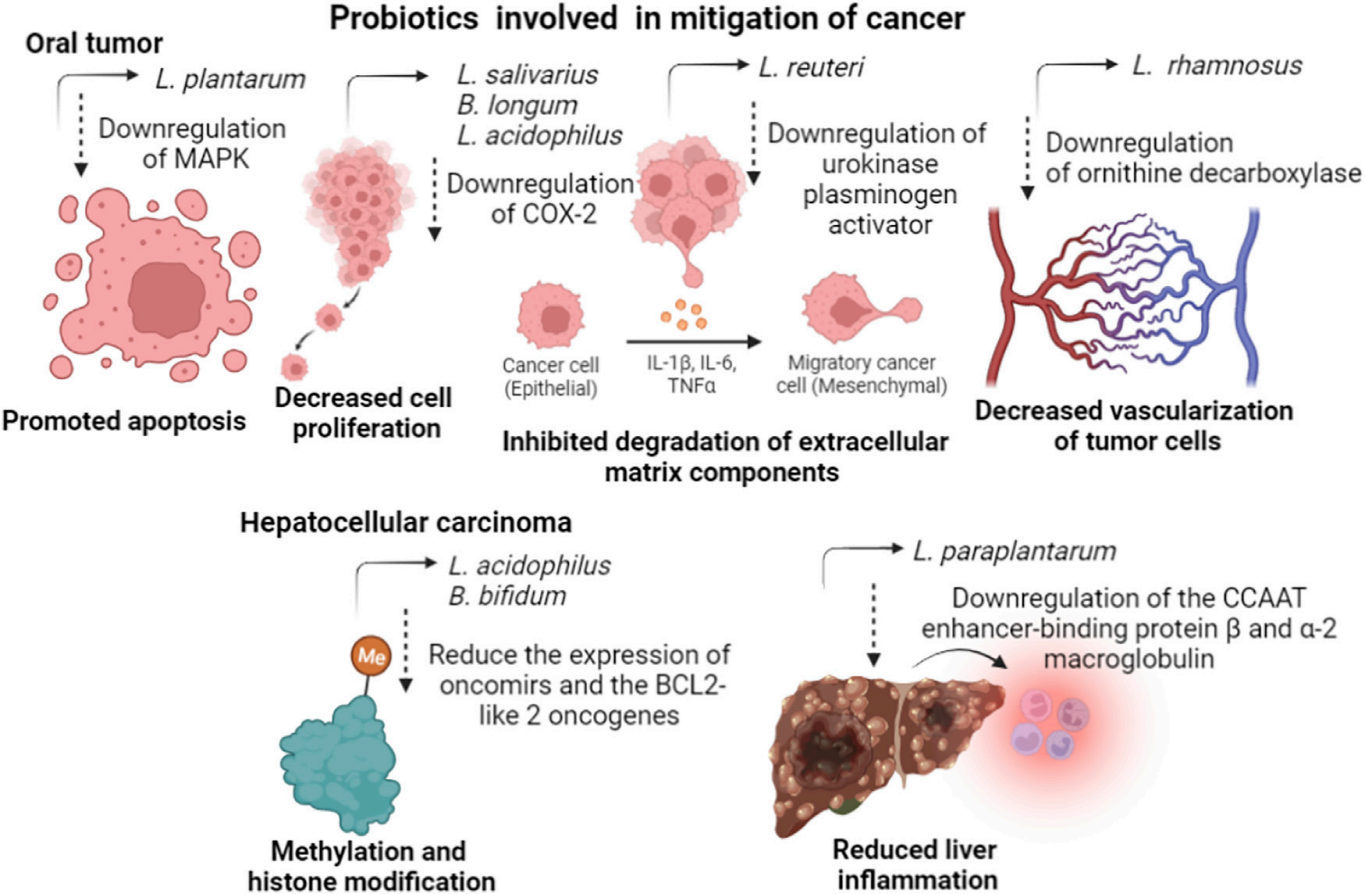
The mechanisms of action of some probiotic strains, cited in this review, in mitigating different types of cancer.

Hepatocellular carcinoma is a type of cancer associated with dysbiosis, and its mitigation can go through treatment with probiotic strains capable of regulating cancer suppressor genes. For example, combinations of different species of probiotic strains can upregulate the expression of anti-inflammatory cytokines, such as interleukins 10, 13, and 27; and downregulation of the angiogenic factors and receptors, vascular endothelial growth factor α, Fms related receptor tyrosine kinase 1, angiopoietin 2, and kinase insert domain receptor, contributing to the reduction of tumor growth. Dysbiosis in the gut microbiome disturbs the gut epithelial integrity and promotes the leakage of opportunistic-associated molecular patterns into the hepatic portal circulation. Reaching the liver promotes inflammation by stimulating the immune cells to produce cytokines and chemokines through Toll-like receptors. In this way, the effects of carcinoma can be attenuated through the downregulation of these genes by the action of probiotic strains (Thilakarathna et al., 2021). Probiotic bacteria *L. acidophilus* and *B. bifidum* can reduce the expression of oncomirs and the oncogenes BCL2-like 2 and Kristen rat viral sarcoma homolog oncogene through methylation and histone modification processes. *L. paraplantarum* reduced liver inflammation and fibrogenesis by downregulating the CCAAT enhancer binding protein *ß* and *a*-2 macroglobulin expressions. The probiotic and prebiotic mixture prevented liver fibrosis by the activation of silent information regulator 1 in hepatocytes. (Thilakarathna et al., 2021). Figure 3 presents a summary of the mechanisms of action of probiotic strains in mitigating different types of cancer.

### Perspectives

The level of knowledge about the mechanisms of action of probiotic strains in the mitigation of different types of cancer has evolved. The use of probiotics, mainly associated with food, was mostly associated with acting on the immune system and decreasing tumor growth, but with less detailed data on the mechanisms. There is already greater detail at the molecular level, mainly in the communication between host cells and the associated microbiomes*via* chemical signaling, particularly in the regulation of host gene expression, as demonstrated in this review article. Probiotic products have already been used as drugs for treating damage caused by chemotherapeutic drugs, such as gastrointestinal mucositis associated with the use of oxaliplatin. Here, the probiotic mixture BIO-THREE, produced by the Toa Pharmaceutical Co., Ltd. containing the bacterial strains *C. butyricum*, *Bacillus mesentericus*, and *Streptococcus faecalis* was used (Yuan et al., 2022). It is to be expected that probiotic strains with more specific action in metabolic pathways and regulation of genes associated with tumor processes will be used as drugs in precision medicine, not only for the large market that this represents, but for the possibility of developing an effective mitigation system of different types of cancer. Table 1 contains a summary of the performance of probiotic strains evaluated in clinical and laboratory trials, with the references cited in this review.

**TABLE 1:** Summary of the performance of probiotic strains evaluated in clinical and laboratory trials, with the references cited in this review.

Cancer	Agent probiotics	Function	Model	References
Colorectal tumor	*Saccharomyces boulardii*	Deregulated pro-inflammatory cytokines	Human	Consoli et al. (2016)
	*Bifidobacterium animalis*	Biogenesis and metabolic pathways of short-chain fatty acids and medium-chain fatty acids related to RNA processing, biosynthesis and metabolism of *a*-amino acids, transmembrane anionic activity, and transferase activity. These actions reduced polyps	Mice	Liao et al. (2021)
	*Lactobacillus plantarum*	Produced gamma-aminobutyric acid and GABAB receptor-dependent signaling pathway, which can be used as a treatment option for 5-fluorouracil-resistant cells because gamma-aminobutyric acid activates antiproliferative, anti-migration, and anti-invasion effects on the resistant cells	Human	An et al. (2021)
	*Bifidobacterium* spp. *Bifidobacterium pseudolongum, Lactobacillus johnsonii Olsenella* spp. *Lactobacillus paracasei* ssp. *Paracasei*. *Bacillus polyfermenticus*	Related to the increased efficacy of immune checkpoint inhibitors	Mice	Wu et al. (2021a)
		Reduction in the expression of genes from the cyclin group of cell cycle regulators associated with tumor development	Human	Davoodvandi et al. (2021)
Melanoma tumor	*Bifidobacterium longum Bifidobacterium breve Bifidobacterium infantis Lactobacillus acidophilus Lactobacillus plantarum Lactobacillus casei Lactobacillus bulgaricus Streptococcus thermophilus*	Related to the production of short-chain fatty acids in the gut, as propionate and butyrate, which promote the expression of chemokine ligand 20 in lung endothelial cells and the recruitment of T helper 17, decreasing the number of tumor foci in lungs	Mice	Chen et al. (2021)
Breast tumor	*Lactobacillus* spp.	Induced specific mechanisms against various infections including cancers through apoptosis, antioxidant activity, immune response, and epigenetics regulation	Human	Laborda-Illanes et al. (2020)
Colon tumor	*Lactobacillus*	Increased apoptosis	Human	Davoodvandi et al. (2021)
	*Lactobacillus casei*	Apoptosis through the upregulated expression of the tumor necrosis factor-related apoptosis-inducing ligand, which was induced by tumor necrosis factor *a*-mediated apoptosis	Human	Davoodvandi et al. (2021)
Gastric tumor	*Lactobacillus* spp.	Inhibited the production of interleukin-8 and interferon gamma, attenuating inflammation in gastric epithelial cells and inhibit the adhesion of the bacterium *Helicobacter pylori,* linked to the initiation of gastric and colorectal cancer	Human	Davoodvandi et al. (2021)
	*Lactobacillus plantarum*	Inhibited the development of cancer cell lines through the downregulation of the Murine Thymoma Viral Oncogene and upregulation of the phosphatase and tensin homolog, B-cell lymphoma 2-associated X, and toll-like receptor 4	Human	Davoodvandi et al. (2021)
Oral tumor	*Lactobacillus plantarum*	Reduced mitogen-activated protein kinase expression and reduced the homeostatic and pathological sequelae caused by intracellular responses under the control of this enzyme	Human	Davoodvandi et al. (2021)
	*Lactobacillus salivarius*	Decreased the expression level of cyclooxygenase-2 and proliferating cell nuclear antigen, decreasing the effects of the disease	Human	Davoodvandi et al. (2021)
	*Lactobacillus rhamnosus*	Inhibited of ornithine decarboxylase and decrease the vascularization of tumor cells	Human	Davoodvandi et al. (2021)
	*Lactobacillus reuteri*	Downregulated the expression level of the urokinase plasminogen activator/urokinase plasminogen activator receptor gene, which is related to the degradation of extracellular matrix components and to cancer metastasis and invasion	Human	Davoodvandi et al. (2021)
	*Bifidobacterium longum Lactobacillus acidophilus*	Expression level of Interleukin-18 was enhanced, inhibiting the proliferation of cancer cells	Human	Davoodvandi et al. (2021)
Hepatocellular carcinoma	*Lactobacillus acidophilus Bifidobacterium bifidum*	Reduced the expression of oncomirs and the oncogenes BCL2-like 2 and Kristen rat viral sarcoma homolog oncogene through methylation and histone modification processes	Human	Thilakarathna et al. (2021)
	*Lactobacillus paraplantarum*	Reduced liver inflammation and fibrogenesis by downregulating the CCAAT enhancer binding protein *ß* and *a*-2 macroglobulin expressions	Human	Thilakarathna et al. (2021)

## Conclusion

Dysbiosis is characterized as imbalances in the diversity of the human microbiome, which can lead to the constitution of different diseases, such as diabetes, allergies, autoimmune, autism, Alzheimer’s, and cancer. Advent such as the use of antibiotics, environmental toxins, processed foods, led to the loss of diversity in the gut microbiome, causing dysbiosis. A consequence of dysbiosis could be the epigenetic inheritances, coordinated by gut microbiome, related to cancer inducing. Therefore, the use of probiotic bacterial strains can recover important intestinal functions and controlling dysbiosis. Furthermore, metabolites of these strains, and of next-generation probiotics, can control epigenetic processes. Bacteria able to improve general human health and to mitigate specific diseases through changes in the regulation of microbiome and host genes are characterized as Live Biotherapeutic Products. These bacteria can induce responses in gut microbiome with implications in chemotherapy, radiotherapy, and immunotherapy, through bacterial metabolism of drugs used in therapeutic. The epigenetic control by probiotic strains or Live Biotherapeutic Products is related to the control of proliferation of cancer cells, inactivation ratios of cancerogenic compounds, improvement of the immune response, antiproliferative effects, inhibition of tyrosine kinase, and inflammatory cell infiltration to malignant and stromal cells. There are still several gaps in studies efficiency of the use of probiotics strains and Live Biotherapeutic Products in cancer treatments because the metabolic interrelationships among resident microbiome, environmental factors and genetic/epigenetic determinants of the vulnerable host are complex. Therefore, metabolome, metagenomics, transcriptome, and analytical chemistry are important approaches to understand the role of bacterial metabolism and chemical communication with cancer predisposition and treatments. The use of probiotics, prebiotics, synbiotics, and metabiotics should be considered as a complement to other more invasive and hazard therapies, such chemotherapy, surgery, and radiotherapy. Thus, it is possible that a path of medicine in the times to come will be more patient-specific treatments, depending on the environmental, genetic, epigenetic and microbiome characteristics of the host.
