# Supplementary material for: Probiotics and live biotherapeutic products aiming at cancer mitigation and patient recover
Source: Front Genet. 2022 Aug 9;13:921972. doi: 10.3389/fgene.2022.921972 (PMC9395637; doi:10.3389/fgene.2022.921972)
Supplement: Supplementary file 1 [file Table1.DOCX]

| **Cancer** | **Agent probiotics** | **Function** | **Model** | **Reference** |
| --- | --- | --- | --- | --- |
| Colorectal tumor | *Saccharomyces boulardii* | Deregulated pro-inflammatory cytokines. | Human | Consoli *et al*., 2016 |
|  | *Bifidobacterium animalis* | Biogenesis and metabolic pathways of short-chain fatty acids and medium-chain fatty acids related to RNA processing, biosynthesis and metabolism of α-amino acids, transmembrane anionic activity, and transferase activity. These actions reduced polyps. | Mice | Liao *et al*., 2021 |
|  | *Lactobacillus plantarum* | Produced gamma-aminobutyric acid and GABAB receptor-dependent signaling pathway, which can be used as a treatment option for 5-fluorouracil-resistant cells because gamma-aminobutyric acid activates antiproliferative, anti-migration, and anti-invasion effects on the resistant cells. | Human | An *et al*., 2021 |
|  | *Bifidobacterium* spp.  *Bifidobacterium pseudolongum*  *Lactobacillus johnsonii Olsenella spp.* | Related to the increased efficacy of immune checkpoint inhibitors. | Mice | Wu *et a*l., 2021 |
|  | *Lactobacillus paracasei* ssp. *paracasei*  *Bacillus polyfermenticus* | Reduction in the expression of genes from the cyclin group of cell cycle regulators associated with tumor development. | Human | Davoodvandi *et al*., 2021 |
| Melanoma tumor | *Bifidobacterium* *longum* *Bifidobacterium* *breve* *Bifidobacterium* *infantis* *Lactobacillus* *acidophilus* *Lactobacillus* *plantarum* *Lactobacillus* *casei* *Lactobacillus* *bulgaricus*  *Streptococcus* *thermophilus* | Related to the production of short-chain fatty acids in the gut, as propionate and butyrate, which promote the expression of chemokine ligand 20 in lung endothelial cells and the recruitment of T helper 17, decreasing the number of tumor foci in lungs. | Mice | Chen *et al*., 2021 |
| Breast tumor | *Lactobacillus* spp. | Induced specific mechanisms against various infections including cancers through apoptosis, antioxidant activity, immune response, and epigenetics regulation. | Human | Laborda-Illanes *et al*., 2020 |
| Colon tumor | *Lactobacillus* | Increased apoptosis. | Human | Davoodvandi *et al*., 2021 |
|  | *Lactobacillus casei* | Apoptosis through the upregulated expression of the tumor necrosis factor-related apoptosis-inducing ligand, which was induced by tumor necrosis factor α-mediated apoptosis. | Human | Davoodvandi *et al*., 2021 |
| Gastric tumor | *Lactobacillus* spp. | Inhibited the production of interleukin-8 and interferon gamma, attenuating inflammation in gastric epithelial cells and inhibit the adhesion of the bacterium *Helicobacter pylori,* linked to the initiation of gastric and colorectal cancer. | Human | Davoodvandi *et al*., 2021 |
|  | *Lactobacillus* *plantarum* | Inhibited the development of cancer cell lines through the downregulation of the Murine Thymoma Viral Oncogene and upregulation of the phosphatase and tensin homolog, B-cell lymphoma 2-associated X, and toll-like receptor 4. | Human | Davoodvandi *et al*., 2021 |
| Oral tumor | *Lactobacillus* *plantarum* | Reduced mitogen-activated protein kinase expression and reduced the homeostatic and pathological sequelae caused by intracellular responses under the control of this enzyme. | Human | Davoodvandi *et al*., 2021 |
|  | *Lactobacillus* *salivarius* | Decreased the expression level of cyclooxygenase-2 and proliferating cell nuclear antigen, decreasing the effects of the disease. | Human | Davoodvandi *et al*., 2021 |
|  | *Lactobacillus rhamnosus* | Inhibited of ornithine decarboxylase and decrease the vascularization of tumor cells. | Human | Davoodvandi *et al*., 2021 |
|  | *Lactobacillus reuteri* | Downregulated the expression level of the urokinase plasminogen activator/urokinase plasminogen activator receptor gene, which is related to the degradation of extracellular matrix components and to cancer metastasis and invasion. | Human | Davoodvandi *et al*., 2021 |
|  | *Bifidobacterium longum*  *Lactobacillus acidophilus* | Expression level of Interleukin-18 was enhanced, inhibiting the proliferation of cancer cells. | Human | Davoodvandi *et al*., 2021 |
| Hepatocellular carcinoma | *Lactobacillus acidophilus*  *Bifidobacterium bifidum* | Reduced the expression of oncomirs and the oncogenes BCL2-like 2 and Kristen rat viral sarcoma homolog oncogene through methylation and histone modification processes. | Human | Thilakarathna *et al*., 2021 |
|  | *Lactobacillus paraplantarum* | Reduced liver inflammation and fibrogenesis by downregulating the CCAAT enhancer binding protein β and α-2 macroglobulin expressions. | Human | Thilakarathna *et al*., 2021 |
